# Epstein-Barr Virus- (EBV-) Immortalized Lymphoblastoid Cell Lines (LCLs) Express High Level of CD23 but Low CD27 to Support Their Growth

**DOI:** 10.1155/2019/6464521

**Published:** 2019-03-28

**Authors:** Hooi-Yeen Yap, Thin-Sam Siow, Sook-Khuan Chow, Sin-Yeang Teow

**Affiliations:** ^1^Department of Medical Sciences, School of Healthcare and Medical Sciences, Sunway University, Jalan Universiti, Bandar Sunway, 47500 Subang Jaya, Selangor Darul Ehsan, Malaysia; ^2^Department of Biological Sciences, School of Science and Technology, Sunway University, Jalan Universiti, Bandar Sunway, 47500 Subang Jaya, Selangor Darul Ehsan, Malaysia; ^3^Sunway Medical Centre, Jalan Lagoon Selatan, Bandar Sunway, 47500 Subang Jaya, Selangor Darul Ehsan, Malaysia

## Abstract

Epstein-Barr virus (EBV) is one of the common human herpesvirus types in the world. EBV is known to infect more than 95% of adults in the world. The virus mainly infects B lymphocytes and could immortalize and transform the cells into EBV-bearing lymphoblastoid cell lines (LCLs). Limited studies have been focused on characterizing the surface marker expression of the immortalized LCLs. This study demonstrates the generation of 15 LCLs from sixteen rheumatoid arthritis (RA) patients and a healthy volunteer using B95-8 marmoset-derived EBV. The success rate of LCL generation was 88.23%. All CD19+ LCLs expressed CD23 (16.94-58.9%) and CD27 (15.74-80.89%) on cell surface. Our data demonstrated two distinct categories of LCLs (fast- and slow-growing) (*p*<0.05) based on their doubling time. The slow-growing LCLs showed lower CD23 level (35.28%) compared to fast-growing LCLs (42.39%). In contrast, the slow-growing LCLs showed higher percentage in both CD27 alone and CD23+CD27+ in combination. Overall, these findings may suggest the correlations of cellular CD23 and CD27 expression with the proliferation rate of the generated LCLs. Increase expression of CD23 may play a role in EBV immortalization of B-cells and the growth and maintenance of the EBV-transformed LCLs while CD27 expression might have inhibitory effects on LCL proliferation. Further investigations are warranted to these speculations.

## 1. Introduction

Epstein-Barr virus (EBV) has infected more than 95% adults globally [1]. EBV mainly targets and infects B-cells followed by epithelial cells and, to a lesser extent, CD4 T-cells [2]. Infectious virions are produced following the lytic replication in B-cells and epithelial cells. After the lytic cycle, EBV latency follows and persists in the infected B-cells for the rest of the individual's life [2]. EBV mainly causes infectious mononucleosis and it is also closely associated with both lymphoid and epithelial malignancies such as Burkitt's lymphoma, Hodgkin's lymphoma, nasopharyngeal carcinoma, and gastric cancer [3].

EBV infects B-cells and could immortalize the B-cells and form long-term growing LCLs which consistently express the viruses [4]. This method has been used in laboratories to immortalize certain human blood-derived B-cells which can be later on used as a culture model for various studies including large-scale drug library screening, vaccine study, and high throughput biological studies [5, 6]. Amongst all, EBV produced from B95-8, a marmoset cell line, appears to be the most common and potent EBV source for B-cell immortalization. In the past decades, researchers have put in effort to improve the immortalization method and its efficiency [7]. Recently, it has been shown that with a minor modification, LCLs could be generated from a small volume of peripheral blood as low as 0.1mL [4]. This finding is particularly useful under a circumstance in which the sample volume is limited.

Previous studies have reported that immune cells particularly B-cells and their subsets play pivotal role in the pathogenesis of RA [8, 9]. Despite the success of EBV immortalization of B-cells, little has been done on the characterization of LCLs and its comparison with the parental B-cells. In this study, we demonstrated that EBV could efficiently immortalize B-cells from RA patients' peripheral blood and form LCLs. We then examined the expression levels of several B-cells subpopulation markers including CD23 and CD27 in both LCLs and their parental B-cells. These B-cell subpopulations have been previously associated with the clinical presentation of RA patients. For instance, elevated B-cells expressing CD23 have been detected in RA patients and they could be blocked by monoclonal antibodies [10]. Similarly, increased amount of CD27^+^IgD^−^ memory B-cells was also readily detected in patients with active RA [11]. On the other hand, Hu and coworkers demonstrated that CD27^+^ B-cells negatively correlated with the disease activity of RA and were found to be impaired in the patients [12].

In this study, we sought to evaluate the expression level of CD23 and/or CD27 of B-cells from the PBMCs and LCLs derived from RA patients. We also investigated the expression level of CD23 and CD27 in regard to the cellular growth of LCLs.

## 2. Materials and Methods

### 2.1. Patient Samples

5mL of peripheral blood from sixteen RA patients (Table 1) was collected from Sunway Medical Centre, Malaysia, under an ethical approval code of 011/2017/ER. PBMCs were isolated using Ficoll-Paque (GE Healthcare) method. Briefly, the blood collected in EDTA tube was diluted 1 to 1 with sterile phosphate-buffered saline (PBS) and layered onto Ficoll-Paque solution in a fresh centrifuge tube. The tube was centrifuged at 400 xg for 40 minutes at room temperature with low acceleration and ‘brake off '. The top layer which is the plasma was collected and aliquoted before storing at -80°C. The buffy coat containing PBMCs was collected using 3mL Pasteur pipette and transferred to a fresh centrifuge tube. The cells were washed twice with PBS followed by RPMI medium containing 10% Fetal Bovine Serum (FBS), before cryopreserved in 10% DMSO and stored in liquid nitrogen storage tank. 5mL of blood was donated from a healthy volunteer as control. The PBMCs and plasma were processed and stored as described above. The date of diagnosis, blood sampling, PBMCs cryopreservation, and EBV immortalization are shown in Supplementary Materials (available here).

### 2.2. Blood Test and Disease Activity Calculation

Blood test result (anti-CCP, RF, ESR, and CRP), tender joint count (TJC), swollen joint count (SJC), and patient global assessment (PtGA) scores of RA patients were retrieved from the rheumatologist's clinic. DAS28-CRP as the indices for disease activity measurement were calculated using the formula (0.56*∗*sqrt(TJC28) + 0.28*∗*sqrt(SJC28) + 0.36*∗*ln(CRP+1) + 0.014*∗*PtGA + 0.96) [13]. The disease activity was classified into remission (<2.6), low (2.6 - 3.1), moderate (3.2 - 5.1), and high (>5.1).

### 2.3. EBV Immortalization

B95-8 marmoset-derived EBV supernatant was a kind gift from Dr. Alan Soo-Beng Khoo, Molecular Pathology unit, Institute for Medical Research (IMR), Malaysia. B-cells from the patient's peripheral blood were immortalized using the concentrated EBV supernatant as previously described [14]. The table in Supplementary Materials shows the date of PBMC isolation and cryopreservation, LCLs establishment, and duration of cryopreserved PBMCs stored in liquid nitrogen storage tank. Briefly, a vial of previously cryopreserved PBMC was thawed out and the cell number was determined. The cells were adjusted to 2 x 10^6^/mL using freshly thawed EBV supernatant and incubated overnight at 37°C, 5% CO_2_ in a standing T25 flask. The next day, transformation medium (RPMI 1640 + 20% FBS + 200ng/ml cyclosporine) was added into the flask. The EBV-infected cells were observed under the microscope to look for rosette-like transformed LCLs in clusters. LCLs in passages 3–6 were used for the flow cytometry analysis and cell proliferation assay.

### 2.4. Flow Cytometry Analysis

Three-colour cytometry was performed on FACSCalibur flow cytometer (Becton Dickinson) using monoclonal antibodies identifying surface antigens for T-cells (CD3/PE, clone SK7), B-cells (CD19/BB515, HIB19), and B-cell subsets (CD23/APC, clone EBV-CS5, and CD27/PE, clone L128). Cell viability solution 7AAD (VIA-PROBE) was purchased to determine the cell viability. All antibodies were purchased from Becton Dickinson. Briefly, cryopreserved PBMCs were thawed and resuspended in 50*µ*L of BSA stain buffer (Becton Dickinson) and incubated at room temperature in the dark with prediluted fluorochrome-conjugated antibodies for 30 minutes. The PBMCs were then washed twice with BSA stain buffer and resuspended in 500*µ*L of BSA buffer for flow cytometry analysis. Control PBMC suspensions were prepared with the same procedure. 20,000 viable cells determined by the cell viability dye were gated and collected for three-colour analysis at sets of CD3/CD19/CD23 and CD19/CD27/CD23. Data were analysed using CellQuest Pro software (Becton Dickinson).

### 2.5. LCLs Proliferation Assay

To separate the LCLs into two categories (fast- and slow-growing), the cell proliferation rate was determined using RealTime-Glo MT cell viability assay (Promega) following the manufacturer's instruction. This is a nonlytic luminescence-based assay that can detect real-time proliferation of live cells in culture up to 72 hours. Briefly, 10,000 cells/well were seeded onto a white flat-bottom 96-well plate (Greiner Bio-one) in triplicate. 2X reaction mix was prepared from the provided kit and added to each well. Cells were incubated at 37°C, 5% CO_2_ for 1 hour before the measurement was taken at different time points (0, 16, 20, 24, 40, 44, 48, 64, 68, and 72 hours) using a luminescence microplate reader (Tecan). The relative luminescence units (RLUs) versus time graph were plotted to show the growth curve of the LCLs. In this graph, the cell number was represented by the RLUs. Using the RLUs, the doubling time (time required for a cell number to double in value) of each category of LCLs was calculated using a multipoint online calculator which were used for significance tests [15]. This online tool uses the least squares fitting exponential method to calculate the doubling time [15]. The growth rate (number of doublings that occur per unit of time) was then calculated using the formula below:

(1) $$\begin{array}{c} Growth\;\;rate=\frac{ln\left(2\right)}{doubling\;\;time} \end{array}$$

The mean value of all LCLs' doubling time was 54.69 hours. Hence, this value was used as the cutoff and LCLs with doubling time higher than 54.69 hours were grouped into slow-growing LCLs and vice versa for fast-growing LCLs.

### 2.6. Statistical Analysis

Flow cytometry analysis was performed in three independent experiments for each category of LCLs, unless otherwise specified. Data were expressed in mean values ± standard deviations (std. dev). Significance was determined using nonparametric tests (Mann-Whitney and Kruskal-Wallis H) in which the data is considered significant when* p *is less than 0.05.

## 3. Results and Discussion

### 3.1. Disease Activity of Study Subjects

Table 1 shows the demographic data of the sixteen RA patients and a healthy individual and their blood test results of commonly used RA diagnostic markers indicating their disease activities. Based on the DAS28-CRP index of 16 RA patients, 3 of these patients were classified as high disease activity, 8 as moderate activity, 3 as remission, and the remaining two as unknown status due to incomplete data collection.

### 3.2. Generation of LCLs

**Figure 1:**
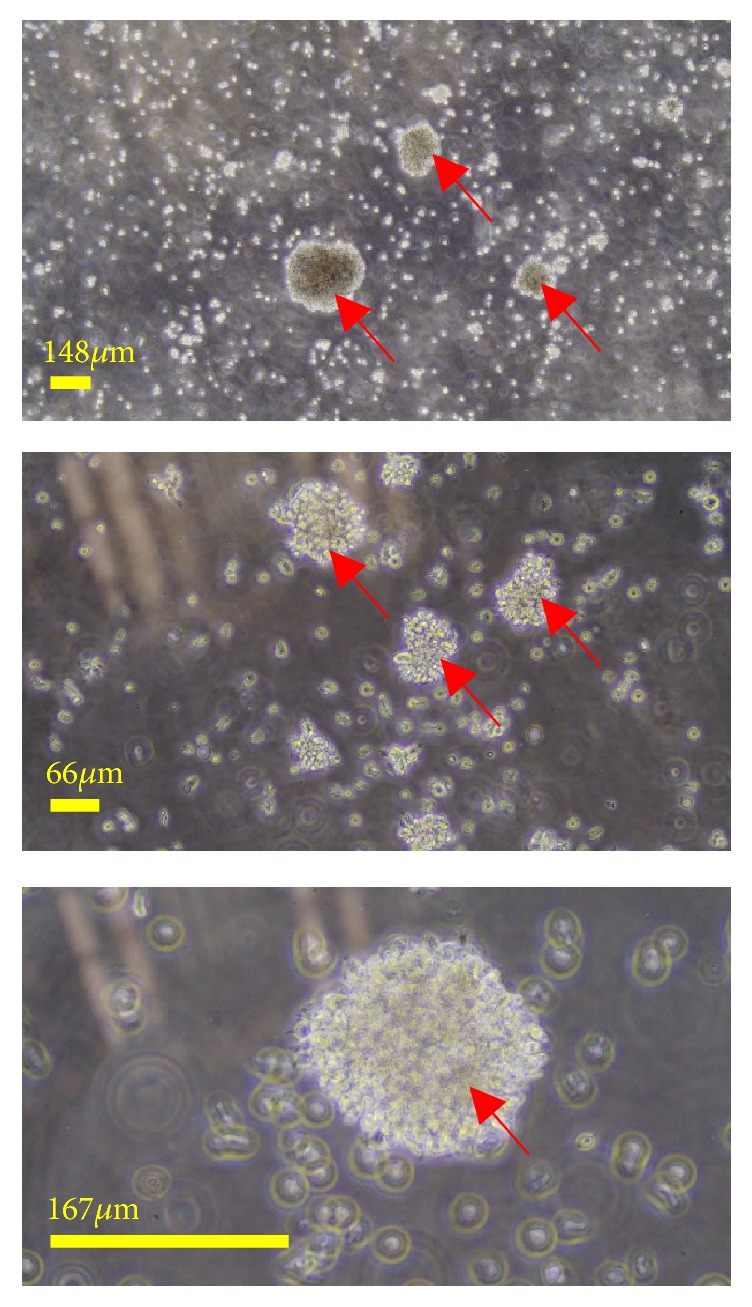
Microscopic examination of PBMC-derived EBV-immortalized B-cells from a RA patient 8A one week after infection. These immortalized cells are annotated as LCL-8A. Red arrows show the rosette-like EBV-immortalized LCLs at 4x (upper panel), 10x (middle panel), and 20x (bottom panel) magnifications. Scale bars are indicated in yellow.

PBMCs were harvested from the sixteen RA patients and one healthy donor (labelled as C) (Table 1) and cryopreserved. Aliquots of cryopreserved PBMCs were then used for EBV infection. Out of the seventeen flasks, 15 EBV-transformed LCLs (except 4E and 14A) were generated one week after infection in which visible cell clusters were seen from the flasks. When visualized under microscope, the LCLs showed typical rosette morphology with various sizes as indicated by the red arrows (Figure 1). The success rate of the LCLs generation was 88.23% (15 out of 17).

### 3.3. Growth Rate and Doubling Time Calculation of Generated LCLs

**Figure 2:**
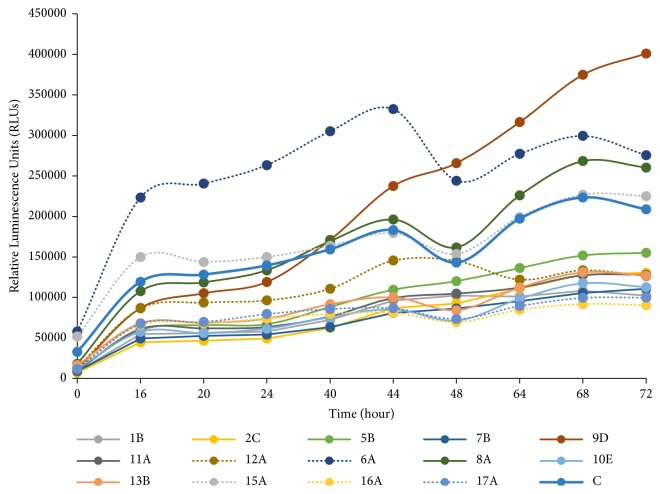
Growth curves of LCLs. Relative luminescence units represent the viability signals of cells at various time points. The filled lines represent the fast-growing LCLs whereas dotted lines represent the slow-growing LCLs.

We then determined the growth rate of each generated category of LCLs using a nonlytic and real-time luminescence-based cell proliferation assay.  Figure 2 shows the growth curve of each category of LCLs represented by the relative luminescence units (RLUs).  Table 2 shows the calculated doubling time and growth rate of respective LCLs using an online calculator [15]. From the obtained doubling time of each category of LCLs, we divide the LCLs into two categories using 54.69 hours (mean value of all LCLs) as the cut-off value, which resulted in 10 fast-growing and 5 slow-growing LCLs (Tables 2 and 4) (*p*<0.05).

### 3.4. Surface Marker Expression of LCLs

**Figure 3:**
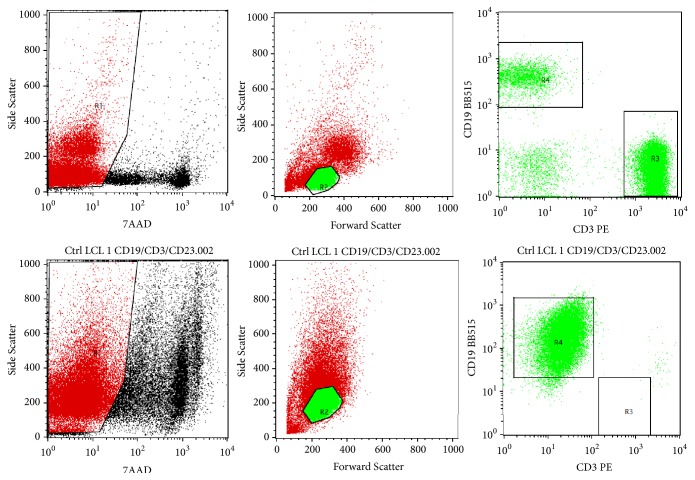
T-cell (CD3) and B-cell (CD19) surface marker profiling by flow cytometry. The upper panel shows the analysis of PBMCs isolated from patient 8A while the lower panel shows the analysis of the corresponding LCLs (LCL-8A).* R1*: 7AAD^−^ live cells;* R2*: gated lymphocytes;* R3*: gated CD3^+^ T-cells;* R4*: gated CD19^+^ B-cells.

PBMCs and corresponding LCLs were subjected to flow cytometry analysis to determine their surface marker expression including CD3, CD19, CD23, and CD27. CD3 and CD19 are surface markers for T-cells and B-cells, respectively. Using the PBMCs isolated from patient 8A and the corresponding LCL-8A as examples, Figure 3 shows how the CD3^+^ T-cells and CD19^+^ B-cells were profiled and gated by flow cytometry. The expression level was then presented in percentage from the total number of analysed lymphocytes which were 20,000 cells. In the donors' PBMCs, 29.78-81.8% were T-cells while 2.35-45.95% comprise B-cells. Following the EBV transformation, T-cell populations reduced to 0-0.27% while B-cell populations increased to 68.59-89.35%. This clearly indicates the success of B-cell immortalization by EBV which has taken over the T-cell population. CD3^+^ T-cells senesced and would be completely washed off from the LCLs.

**Figure 4:**
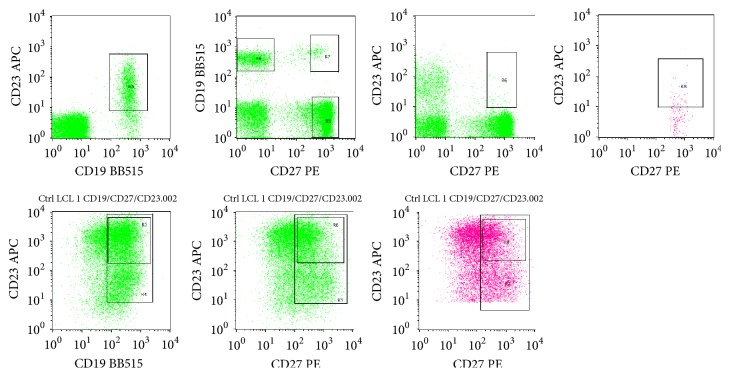
CD23 and CD27 expression analysis of CD19^+^ B-cells by flow cytometry. The upper panel shows the analysis of PBMCs isolated from patient 8A while the lower panel shows the analysis of the corresponding LCLs (LCL-8A).* R3*: gated CD27^+^ cells;* R4*: gated CD19^+^ B-cells;* R5*: gated CD19^+^CD23^+^;* R6*: gated CD23^+^CD27^+^ cells;* R7*: gated CD19^+^CD27^+^ cells;* R8*: gated CD19^+^CD23^+^CD27^+^ cells.

CD23 is a B-cell activation marker while CD27 is a marker for memory B-cells [16, 17]. In RA patients, elevated levels of CD23-expressing B-cells have been previously reported to positively correlate with the disease activity, and they could potentially serve as a therapeutic target [10, 18]. In contrast, CD27^+^ B-cells were found to negatively correlate with disease activity and found to be impaired in RA patients [12]. In the current study, we could not differentiate the expression pattern of CD23 and CD27 in PBMCs and LCLs (Table 3).  Figure 4 depicts the gating of CD19^+^, CD23^+^, and CD27^+^ from the PBMCs isolated from patient 8A and the corresponding LCL-8A by flow cytometry. From all LCLs, while 68.59-89.35% were CD19^+^ as expected, they exhibit expression of CD23 (16.94-58.9%) and CD27 (15.74-80.89%). B-cells coexpressing CD23 and CD27 were 5.72-41.53% (Table 3).

### 3.5. Correlation of Disease Activity, Surface Marker Expression Level, and LCL Proliferation

A statistical analysis was performed to test the correlation between the disease activity and surface marker expressions; however, none of them showed significance. Similarly, the disease activity did not correlate with the growth rate of corresponding LCLs.

From the doubling time of each category of LCLs, mean values for both fast- and slow-growing LCLs were determined and subjected to statistical analysis.  Table 4 shows a significant difference (*p*<0.05) in terms of doubling time between the two categories. Similarly, the mean values of the CD23, CD27, and CD23+CD27+ levels of the respective category were determined. The fast-growing LCLs have higher expression of CD23 (42.39%) compared to the slow-growing LCLs (35.28%). However, this is not statistically significant (Table 4). Inversely, CD27 expression and CD23/CD27 coexpression were lower in the fast-growing LCLs (36.15 and 13.36%, respectively) compared to the slow-growing LCLs (51.57% and 19.49%, respectively). When a statistical analysis is performed, only the fast-growing LCLs show significantly lower in CD27 but not CD23/CD27 expression. Further studies with larger sample size are required to validate these findings.

The high expression of CD23 might be due to the EBV infection or transformation. There is evidence showing that B-cell immortalization might rely on the cellular CD23 expression. The study showed that CD23-negative B-cells could be infected by EBV but could not be immortalized unless supplementing with specific growth factors [19]. Another study showed that EBV-infected B-cells could develop into two distinct subpopulations: CD23^high^CD58^+^IL6^−^ and CD23^low^CD58^+^IL6^+^ [20]. The former B-cell subpopulation actively proliferated while the latter subpopulation ceased to proliferate. The expression of CD23 might also be induced by EBV nuclear antigen 2 (EBNA-2) expression following the EBV transformation of B-cells as previously described in a Burkitt lymphoma (BL) cell line [16]. In contrast, the higher expression of CD27 in slow-growing LCLs may suggest their inhibitory effects on the LCLs' proliferation.

## 4. Conclusions

CD23 could play a stimulatory role in EBV transformation of B-cells and the LCLs' growth. Our studies showed that the fast-growing LCLs exhibited higher CD23 expression and lower CD27 expression and CD23CD27 expression. Further studies with a larger sample size are warranted to investigate in depth on the implication of the surface marker expression on the EBV maintenance and cellular growth.

## Figures and Tables

**Table 1:** Patient's demographic data, blood test result, and success of LCLs generation.

Sample annotation	Age	Gender	Ethnic	Anti-CCP (IU/mL)	RF (IU/mL)	ESR (mm/hr)	CRP (mg/L)	DAS28-CRP	Disease activity	LCL generation
1B	71	Female	Chinese	269.7	70	11	4.9	2.02	Remission	Yes
2C	60	Male	Indian	58.8	15	66	81.4	5.96	High	Yes
4E	66	Female	Chinese	91.2	143	12	4.3	2.09	Remission	No
5B	77	Female	Chinese	256	69	4	12.1	2.54	Remission	Yes
6A	64	Female	Chinese	515.7	101	44	12.1	4.19	Moderate	Yes
7B	54	Female	Chinese	16	304	42	15.1	3.92	Moderate	Yes
8A	50	Male	Chinese	249.3	68	46	15.2	6.59	High	Yes
9D	69	Male	Chinese	2256	409	5	3.7	-	-	Yes
10E	70	Female	Chinese	2.9	15	42	23.7	-	-	Yes
11A	38	Female	Chinese	60.7	17	16	1.4	4.22	Moderate	Yes
12A	63	Female	Malay	<0.5	<10	74	77	5.99	High	Yes
13B	50	Female	Chinese	179.7	462	81	8.3	4.63	Moderate	Yes
14A	36	Female	Indian	64.1	83.4	66	13.6	3.81	Moderate	No
15A	55	Male	Chinese	849.2	137	10	2	3.41	Moderate	Yes
16A	62	Female	Chinese	849.2	137	7	20.8	4.49	Moderate	Yes
17A	36	Female	Chinese	2.6	55	18	13.8	4.47	Moderate	Yes
C	29	Male	Chinese	-	-	-	-	-	-	Yes

Anti-CCP: anti-cyclic citrullinated peptide antibody; CRP: C-reactive protein; DAS28: disease activity score 28; ESR: erythrocyte sedimentation rate; RF: rheumatoid factor.

**Table 2:** Doubling time and growth rate of LCLs.

Sample	Doubling time (hour)	Growth rate	Category
1B	47.16	0.0147	Fast
2C	31.69	0.0219	Fast
5B	36.05	0.0192	Fast
6A	107.32	0.0065	Slow
7B	41.4	0.0167	Fast
8A	39.69	0.0175	Fast
9D	25.92	0.0267	Fast
10E	44.57	0.0156	Fast
11A	42.48	0.0163	Fast
12A	73.59	0.0094	Slow
13B	48.74	0.0142	Fast
15A	64.14	0.0108	Slow
16A	84.93	0.0082	Slow
17A	78.53	0.0088	Slow
C	54.2	0.0128	Fast

**Table 3:** Surface marker expression of PBMCs and LCLs derived from participated subjects.

Sample type and annotation		Surface marker expression (%) ± standard deviation				
		CD3+	CD19+	CD19+CD23+	CD19+CD27+	CD19+CD23+CD27+
1B	PBMC-1B	66.58	5.62	3.92	0.36	0.01
	LCL-1B	0.02	81.19 ± 0.77	49.26 ± 1.26	17.75 ± 1.51	6.63 ± 0.93
2C	PBMC-2C	78.72	6.07	5.57	1.56	0.41
	LCL-2C	0.06 ± 0.03	86.61 ± 0.38	39.57 ± 0.25	53.66 ± 0.56	20.59 ± 0.31
5B	PBMC-5B	44.10	14.02	7.97	3.73	0.25
	LCL-5B	0.09 ± 0.05	79.47 ± 0.31	38.52 ± 0.16	51.5 ± 1.03	19.09 ± 0.68
6A^#^	PBMC-6A	-	19.4	19.5	3.7	1.0
	LCL-6A^#^	-	77.4	27.4	39.7	11.06
7B	PBMC-7B	72.27	10.95	8.70	1.49	0.22
	LCL-7B	0.01 ± 0.02	80.58 ± 1.28	48.06 ± 1.29	23.34 ± 5.76	8.79 ± 3.13
8A	PBMC-8A	66.6	16.2	17.9	2.34	1.24
	LCL-8A	0.14 ± 0.21	86.0 ± 0.83	58.9 ± 2.58	34.4 ± 4.90	17.6 ± 1.37
9D	PBMC-9D	49.57	2.56	2.06	0.18	0.03
	LCL-9D	0.02 ± 0.01	71.32 ± 0.87	43.17 ± 0.70	15.74 ± 0.28	5.72 ± 0.22
10E	PBMC-10E	34.15	23.58	22.57	3.89	0.64
	LCL-10E	0	76.59 ± 0.51	44.72 ± 0.24	43.94 ± 0.03	19.54 ± 0.18
11A	PBMC-11A	59.45	13.02	8.75	3.74	0.57
	LCL-11A	0.05 ± 0.01	68.59 ± 0.73	16.94 ± 0.37	48.83 ± 1.10	8.89 ± 0.30
12A	PBMC-12A	48.27	9.10	7.58	5.42	0.53
	LCL-12A	0.05 ± 0.01	72.70 ± 1.20	18.29 ± 0.40	51.64 ± 1.11	9.73 ± 0.21
13B	PBMC-13B	29.78	45.95	13.61	1.65	0.10
	LCL-13B	0.01 + 0.01	74.95 + 1.42	52.87 + 1.54	20.18 + 1.51	12.74 + 1.2
15A	PBMC-15A	10.46	2.35	1.93	1.12	0.11
	LCL-15A	0	88.84 + 1.72	19.47 + 0.46	80.89 + 0.82	17.78 + 0.49
16A	PBMC-16A	81.8	9.66	9.74	2.66	0.82
	LCL-16A	0.27 ± 0.41	72.8 ± 0.64	43.7 ± 1.81	50.6 ± 8.63	26.0 ± 3.05
17A	PBMC-17A	69.28	11.64	10.66	3.39	0.61
	LCL-17A	0	89.35 + 1.03	41.15 + 2.07	80.06 + 1.59	41.53 + 0.65
C	PBMC-C	75.5	16.3	15.4	1.85	0.59
	LCL-C	0.10 ± 0.14	70.6 ± 1.73	44.1 ± 4.04	37.9 ± 0.24	17.6 ± 1.80

LCL: lymphoblastoid cell line; PBMC: peripheral blood mononuclear cell.

^#^Flow cytometric analysis for all surface markers was only performed once and CD3 levels were not determined due to the limitation of cell number.

**Table 4:** Statistical analysis of CD23, CD27, and CD23CD27 expression of fast- and slow-growing LCLs.

Category	Doubling time		Growth rate		CD23 (%)		CD27 (%)		CD23 CD27 (%)	
	mean	*p *value	mean	*p *value	mean	*p *value	mean	*p *value	mean	*p *value
Fast (*n* – 10)	38.62	0.001	0.0186	0.001	42.39	0.075	36.15	0.028	13.36	0.241
Slow (*n* – 5)	73.06		0.0101		35.28		51.57		19.49	

## Data Availability

The data used to support the findings of this study are included within the article.

## Supplementary Materials

Supplementary Materials The table shows the studied cases with date of diagnosis, blood sampling, PBMCs cryopreservation, EBV immortalization, and duration of cryopreserved PBMCs.
