# Supplementary material for: Epstein-Barr Virus- (EBV-) Immortalized Lymphoblastoid Cell Lines (LCLs) Express High Level of CD23 but Low CD27 to Support Their Growth
Source: Adv Virol. 2019 Mar 28;2019:6464521. doi: 10.1155/2019/6464521 (PMC6458955; doi:10.1155/2019/6464521)
Supplement: Supplementary Materials — The table shows the studied cases with date of diagnosis, blood sampling, PBMCs cryopreservation, EBV immortalization, and duration of cryopreserved PBMCs. [file 6464521.f1.docx]

**Supplemental data:**

**Supplemental Table 1:**

| **Sample** | **Date of diagnosis** | **Date of blood sampling and PBMC cryopreservation** | **Date of PBMC revival and EBV immortalization** | **Duration of PBMC cryopreservation (days)** |
| --- | --- | --- | --- | --- |
| 1B | 21^st^ Aug 2017 | 24^th^ Nov 2017 | 18^th^ Jan 2019 | 421 |
| 2C | 14^th^ Sept 2017 | 15^th^ Mar 2018 | 18^th^ Jan 2019 | 310 |
| 4E | 11^th^ Oct 2017 | 13^th^ Sept 2018 | 10^th^ Jan 2019 | 120 |
| 5B | 16^th^ Oct 2017 | 30^th^ Apr 2018 | 18^th^ Jan 2019 | 264 |
| 6A | 27^th^ Oct 2017 | 27^th^ Oct 2017 | 11^th^ Aug 2018 | 289 |
| 7B | 10^th^ Nov 2017 | 6^th^ Feb 2018 | 18^th^ Jan 2019 | 347 |
| 8A | 23^rd^ Nov 2017 | 23^rd^ Nov 2017 | 11^th^ Aug 2018 | 262 |
| 9D | 6^th^ Dec 2017 | 24^th^ Sept 2018 | 10^th^ Jan 2019 | 109 |
| 10E | 8^th^ Dec 2017 | 21^st^ Sept 2018 | 10^th^ Jan 2019 | 112 |
| 11A | 13^th^ Dec 2017 | 13^th^ Dec 2017 | 18^th^ Jan 2019 | 402 |
| 12A | 20^th^ Dec 2017 | 20^th^ Dec 2017 | 18^th^ Jan 2019 | 395 |
| 13B | 28^th^ Mar 2018 | 25^th^ June 2018 | 10^th^ Jan 2019 | 200 |
| 14A | 18^th^ Apr 2018 | 18^th^ Apr 2018 | 11^th^ Aug 2018 | 116 |
| 15A | 20^th^ Apr 2018 | 20^th^ Apr 2018 | 10^th^ Jan 2019 | 266 |
| 16A | 20^th^ Apr 2018 | 20^th^ Apr 2018 | 11^th^ Aug 2018 | 114 |
| 17A | 23^rd^ Apr 2018 | 23^rd^ Apr 2018 | 10^th^ Jan 2019 | 263 |
| C | - | 8^th^ May 2018 | 11^th^ Aug 2018 | 96 |
